# TRC-PAD: Accelerating Recruitment of AD Clinical Trials through Innovative Information Technology

**DOI:** 10.14283/jpad.2020.48

**Published:** 2020

**Authors:** G.A. Jimenez-Maggiora, S. Bruschi, R. Raman, O. Langford, M. Donohue, M.S. Rafii, R.A. Sperling, J.L. Cummings, P.S. Aisen, the TRC-PAD Investigators

**Affiliations:** 1. Alzheimer’s Therapeutic Research Institute, University of Southern California, San Diego, CA, USA; 2. Center for Alzheimer Research and Treatment, Brigham and Women’s Hospital, Harvard Medical School, Boston, MA, USA; 3. Department of Brain Health, School of Integrated Health Sciences, University of Las Vegas, Nevada; Cleveland Clinic Lou Ruvo Center for Brain Health, USA

**Keywords:** Alzheimer’s, clinical trials, informatics, recruitment

## Abstract

**BACKGROUND:**

The Trial-Ready Cohort for Preclinical/Prodromal Alzheimer’s Disease (TRC-PAD) Informatics Platform (TRC-PAD IP) was developed to facilitate the efficient selection, recruitment, and assessment of study participants in support of the TRC-PAD program.

**OBJECTIVES:**

Describe the innovative architecture, workflows, and components of the TRC-PAD IP.

**DESIGN:**

The TRC-PAD IP was conceived as a secure, scalable, multi-tiered information management platform designed to facilitate high-throughput, cost-effective selection, recruitment, and assessment of TRC-PAD study participants and to develop a learning algorithm to select amyloid-bearing participants to participate in trials of early-stage Alzheimer’s disease.

**SETTING:**

TRC-PAD participants were evaluated using both web-based and in-person assessments to predict their risk of amyloid biomarker abnormalities and eligibility for preclinical and prodromal clinical trials. Participant data were integrated across multiple stages to inform the prediction of amyloid biomarker elevation.

**PARTICIPANTS:**

TRC-PAD participants were age 50 and above, with an interest in participating in Alzheimer’s research.

**MEASUREMENTS:**

TRC-PAD participants’ cognitive performance and subjective memory concerns were remotely assessed on a longitudinal basis to predict participant risk of biomarker abnormalities. Those participants determined to be at the highest risk were invited to an in-clinic screening visit for a full battery of clinical and cognitive assessments and amyloid biomarker confirmation using positron emission tomography (PET) or lumbar puncture (LP).

**RESULTS:**

The TRC-PAD IP supported growth in recruitment, screening, and enrollment of TRC-PAD participants by leveraging a secure, scalable, cost-effective cloud-based information technology architecture.

**CONCLUSIONS:**

The TRC-PAD program and its underlying information management infrastructure, TRC-PAD IP, have demonstrated feasibility concerning the program aims. The flexible and modular design of the TRC-PAD IP will accommodate the introduction of emerging diagnostic technologies.

## Background

Alzheimer’s Disease (AD) has emerged as one of the most significant public health issues of the 21st century. In 2020, it was estimated that 5.8 million people in the United States (U.S.) were living with Alzheimer’s Disease; this number was expected to rise to 13.8 million by 2050 (1). The development of effective disease-modifying interventions for Alzheimer’s disease (AD) remains an enormously important world health need. As therapeutic research has expanded to early-stage interventions, the recruitment of minimally affected optimally selected study participants has been challenging (2). The challenges include: identifying potential asymptomatic participants with elevated amyloid levels who meet study criteria, expanding recruitment and participation of groups that have been traditionally underrepresented in AD clinical trials, and reducing the time and cost of screening failures (3). Novel approaches to improve participant selection and recruitment using sophisticated informatics platforms have been developed (4, 5). The Trial-Ready Cohort for Preclinical/Prodromal Alzheimer’s Disease (TRC-PAD) program was initiated with the overarching goal of accelerating therapeutic development for AD through the establishment of an infrastructure to ensure timely recruitment of targeted individuals into optimally designed trials (6). More specifically, TRC-PAD aimed to establish a biomarker-confirmed trial-ready cohort to facilitate recruitment into preclinical and prodromal AD trials by using an efficient, low-cost multi-stage selection process driven by an adaptive risk algorithm which combined web-based and in-person longitudinal assessment of participants. The TRC-PAD Informatics Platform (TRC-PAD IP) was developed to facilitate the selection, recruitment, and assessment of study participants in support of the TRC-PAD program. The purpose of this article is to describe the innovative architecture, workflows, and components of the TRC-PAD IP, demonstrate its utility, and discuss broader implications for the field of AD clinical trials.

## Methods

### TRC-PAD Informatics Platform

**Figure 1.**
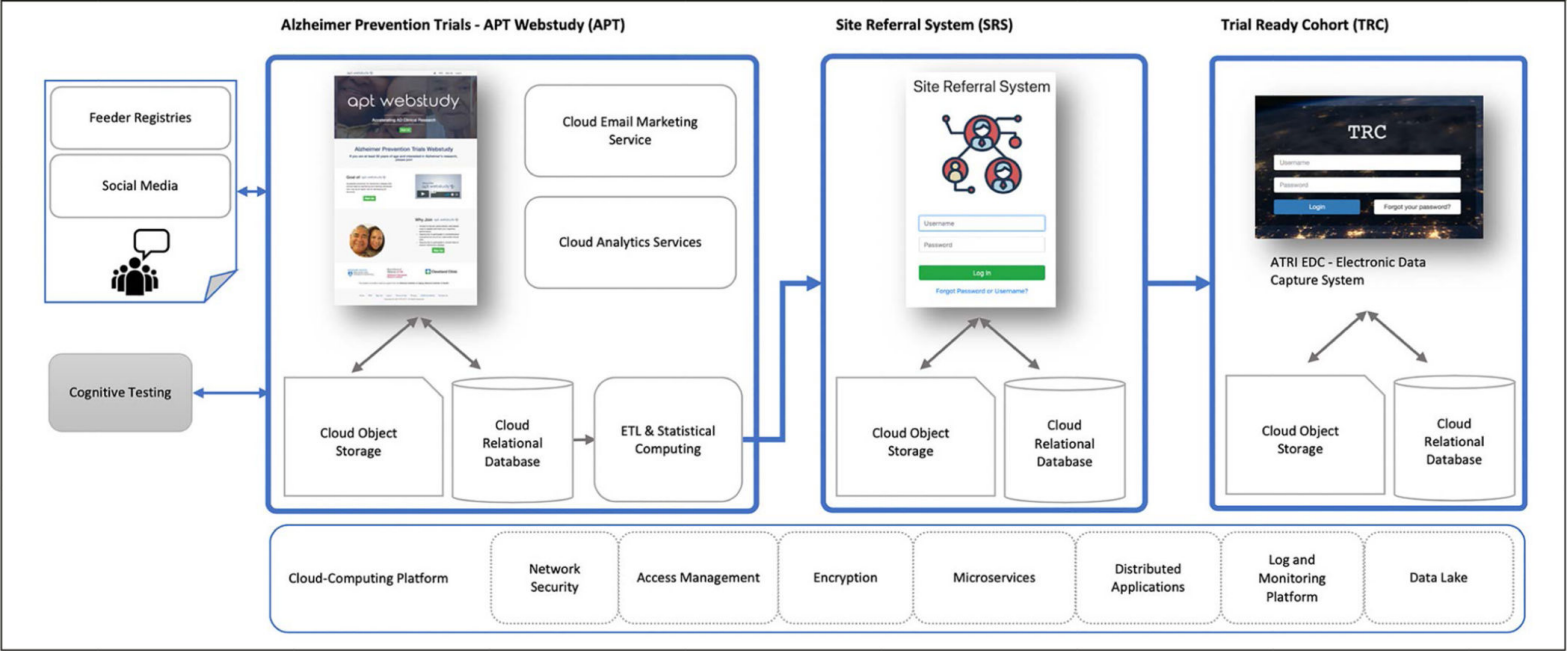
TRC-PAD Informatics Platform

The TRC-PAD Informatics Platform (TRC-PAD IP) (Figure 1) was conceived as a secure, scalable, multi-tiered information management platform designed to facilitate high-throughput, cost-effective selection, recruitment, and assessment of TRC-PAD study participants. The program aims called for the construction of a multi-stage process that encompassed 1) a public-facing web-based registry, the Alzheimer Prevention Trials (APT) Webstudy registry, 2) an analytics platform capable of supporting the development and implementation of risk-based referral and screening algorithms, 3) a web-based referral management system, the Site Referral System (SRS), and 4) a regulatory-compliant clinical data management system to collect data for the standing Trial Ready Cohort (TRC). Given the complex structure and pathways of this process, tracking participants as they entered the program and transitioned from one stage to the next was also a critical requirement. The component systems underpinning this process are described in the following sections.

### APT Webstudy

The APT Webstudy (www.aptwebstudy.org) is a public-facing online longitudinal study that invited members of the general population who were over 50 years of age and had sufficient interest in participating in AD research to register for a user account. Once registered, users were presented with a guided walkthrough during which they provided their contact information and preferences, current AD-related diagnosis (if known), and interest in participating in AD-related research. Subsequently, users were offered additional study information, asked to consider the benefits and risks of study participation, and allowed to consent and enroll in the APT Webstudy using an online consent process. Participants were subsequently asked to provide additional information regarding their demographics, family and medical history, lifestyle, current AD biomarker status (if know), and complete assessments of their subjective memory perceptions using the Cognitive Function Instrument (CFI), and cognitive performance using the Cogstate Brief Battery (7–9). To minimize participant burden, the registration, consent, and assessment process was designed to take approximately 15 to 20 minutes to complete. Follow-up assessments, scheduled every 3 months, were designed to be completed in 10 to 15 minutes to promote participant retention.

### Website Design

The APT Webstudy was designed with several features to support usability and engagement. Users had the option to create their accounts using a «1-click» social login instead of a traditional approach which requires a username, password, and email address. Participants were provided with a dashboard to view their previous assessment scores. They could also download a report with all their information and assessment scores. Participants received a quarterly newsletter via electronic mail developed by leading AD researchers which highlighted new developments in the field as well as news related to the TRC-PAD program. The website was designed to support multiple screen sizes (phone, tablet, desktop) using responsive web design principles (10).

### Longitudinal Assessments

To improve the prediction of participant risk, the APT Webstudy was designed to incorporate unsupervised cognitive assessments as part of a longitudinal observational study. Thus, participants are invited to return to the website every 3 months to complete a new round of assessments and update their information as needed. These follow-up assessments are designed to take 10 to 15 minutes to complete. To encourage compliance, participants received quarterly electronic mail reminders to log-in to the APT Webstudy website to complete their web-based assessments.

### Website Architecture

An important consideration in a public-facing website is the variability of website traffic patterns. Websites with highly volatile traffic patterns can exhibit occasional periods of poor performance or unavailability. This is especially true when an unexpected media event, such as a viral social media mention or a celebrity endorsement, draws a burst of traffic toward the website. To mitigate the effects of these unexpected traffic spikes, the APT Webstudy has an architecture that dynamically adjusts its capacity to respond to incoming requests as changes in traffic patterns emerge. This architecture combines a cloud-based fleet of webservers, a multi-region database cluster, and a dynamic networking configuration to allow the website to automatically activate and deactivate computational resources on-demand to maintain performance during high-traffic periods in a cost-effective manner.

### Recruitment and Retention

Recruitment into the APT Webstudy relied on a combination of traditional and digital media strategies. These efforts included community-based events, local and national paid media campaigns, social media campaigns, and earned media. Additionally, a group of preexisting registries which included the Brain Health Registry (brainhealthregistry.org), the Alzheimer’s Prevention Registry (endalznow.org), Trial Match (trialmatch.alz.org), and Healthy Brains (healthybrains.org), partnered with the TRC-PAD program to refer participants from their cohorts. These registries, collectively known as the “feeder” registries, provided the APT Webstudy with access to a large, engaged group of potential participants. The performance of these recruitment efforts was tracked by using Urchin Tracking Module (UTM) codes which associated participants with the campaign that referred them to the APT Webstudy (11).

### Participant Technical Support

APT Webstudy participants were provided with both telephone- and email-based support channels. Telephone-based support requests were automatically transcribed and forwarded to the email-based support management system for follow-up. This architecture allowed the APT Webstudy support team to manage all incoming support requests via a single interface. Support cycle times and participant satisfaction were among the Key Performance Indicators (KPIs) used to assess and manage the performance of the support team.

### Site Referral System

APT Webstudy participant data were regularly evaluated using an adaptive algorithm that assessed each participant’s risk of AD biomarker positivity and ranked participants based on predicted risk (12). Participants determined to have the highest risk were referred to the nearest TRC-PAD performance site based on their self-reported 5-digit Zip Code. Participant referrals were provided to performance sites via the Site Referral System (SRS), a secure website that allowed authorized site personnel to manage the site’s referral queue and ensure the timely disposition of each referral. Using the SRS, site personnel contacted participants and invited them to schedule an initial in-person screening visit to determine their eligibility for enrollment into the TRC-PAD standing cohort.

### Website Design

A key concept guiding the design of the SRS was the idea that site referral queues would be managed collaboratively among multiple site personnel. To support collaboration, the SRS website incorporated several features that optimized team-based management and disposition of participant referrals. For example, as new referrals became available in the referral queue, site personnel received notifications via electronic mail. The status of every referral in the queue was summarized in a dashboard view facilitating management and reporting. Each referral provided site personnel with access to a summary of participant-reported demographic, medical history, lifestyle, and contact information, as well as status changes. As a final step, referrals were assigned an outcome code and marked complete.

### Trial Ready Cohort

The TRC was conceived as the final stage in the TRC-PAD process. The initial target for the TRC was to enroll a standing longitudinal cohort of 2,000 biomarker-confirmed participants (50% preclinical and 50% prodromal) (6). An Electronic Data Capture (TRC EDC) system was developed to manage TRC participant data, based on the Alzheimer’s Therapeutic Research Institute (ATRI) EDC system. The TRC EDC was used to operationalize a multi-stage screening process and collect a rich set of clinical, neuropsychological, neuroimaging, and biospecimen data collected in a multi-site setting. The TRC EDC was validated to comply with CRF Title 21 Part 11 (13). Doing so allowed the TRC data to be eligible for use as run-in data in downstream clinical trials.

### TRC EDC Design

The TRC EDC was built to provide centralized management of all critical cohort study dataflows and workflows. This approach provided study teams with broad transparency and management capabilities over the study’s complex dataflows and processes. Implementing this approach, however, has proven challenging for traditional systems, which struggle to scale up as larger and more complex data types are introduced. Historically, a solution to this problem has been the implementation of interconnected purpose-specific systems. While feasible, this solution suffers from several shortcomings: 1) supporting evolving study requirements requires complex multi-system impact analysis, 2) training on multiple systems increases the burden on study teams, and 3) data integrations require ongoing maintenance as systems are updated. The TRC EDC was designed to avoid these issues by implementing a cloud-native architecture, which allowed it to harness the full range of capabilities of the underlying cloud platform on which it was hosted. In this architecture, the TRC EDC served as an orchestration engine that coordinated and delegated computational workloads, such as uploading and processing large binary objects (e.g. medical images, sensor data, multimedia data, genetic data, graph data), to the underlying cloud-based service best-suited for the required function, without impacting the study team’s interaction with the system.

### Site Network

An initial step toward the establishment of the TRC was the creation of the TRC-PAD site network, a set of 35 academic clinical sites distributed across the large population centers in the contiguous United States. Sites were selected based on multiple criteria including study team research experience and expertise, amyloid positron emission tomography (PET) imaging and radiotracer availability, and track record in AD/AD and related disorders (ADRD) clinical trials. Site selection and activation were conducted in two stages, the “vanguard” or initial phase, which included 8 pilot sites, and the broad activation phase. The vanguard phase was used as a learning exercise to test and refine TRC-PAD data management tools and processes. These learnings were applied during the broad activation phase to ensure the full site network was optimized.

### Multi-stage Selection and Screening Process

The TRC-PAD selection and recruitment processes were managed by a series of learning algorithms that assessed participant data at multiple points. Newly acquired data were used to update participant risk predictions and rankings (12). These results informed the decision-making process used to graduate participants from one stage of TRC-PAD to the next, culminating with the final determination on enrollment eligibility into the standing TRC.

### Referral to Downstream Clinical Trials

TRC participants who meet eligibility criteria will be referred for screening and potential enrollment to downstream clinical trials, temporarily suspending additional longitudinal assessments and data collection activities in the TRC EDC. In these cases, a link between a participant’s TRC data record and their downstream clinical trial data record will be established via the use of the National Institute on Aging’s (NIA) Global Unique Identifier (GUID) (14, 15). This link will allow for participant data to be integrated across cohorts and used to further inform the TRC-PAD learning algorithms.

### Analytical Platform

TRC-PAD IP supported a single Analytical Platform (AP) that aggregated data from multiple sources in a single semi-structured repository, also known as a «data lake» (16). This approach combined the use of serverless computing methods with fast, low-cost object storage to facilitate the development and operationalization of multiple analysis workloads such as machine learning algorithms, statistical analyses, and reports.

### Information Architecture

The TRC-PAD IP was constructed using open source web development and scientific computing tools hosted on Amazon Web Services (AWS), a public cloud computing platform (Table 1). The TRC-PAD program partnered with AWS’s consulting group, AWS Professional Services (AWS ProServ), to construct a secure, scalable, cost-effective computational infrastructure. The component systems of the TRC-PAD IP were built using a phased approach. Unique system-generated identification numbers (IDs) were assigned to each participant as they moved from one stage of TRC-PAD to the next. These IDs were linked across component systems to maintain the integrity of each participant’s data record while protecting confidentiality.

### Scalability and Cost

The TRC-PAD IP was built on cloud-based computational infrastructure that was optimized to support each component system. The infrastructure supporting the APT Webstudy, which was subject to temporary bursts of traffic due to recruitment campaigns or unexpected media events, was designed to dynamically adapt to changing web traffic patterns by increasing or decreasing its fleet of webservers, within set cost parameters. Likewise, the TRC EDC, which managed a large, regulatory-compliant cohort database, used a cost-effective, highly durable storage strategy. In this architecture, computational resources were used on-demand, reducing idle capacity and providing flexibility as the TRC-PAD program’s requirements evolved.

### Security and Compliance

Ensuring participant confidentiality and data security were the primary requirements for the TRC-PAD IP. Thus, the TRC-PAD Informatics team worked with AWS ProServ to build a Health Insurance Portability and Accountability Act (HIPAA)-eligible architecture and implement AWS’s best practices (17). This architecture was designed to take advantage of multiple strategies to ensure data security and durability such as a multi-account structure, multi-region, encrypted data storage, and automated policy management. To further ensure data security, the TRC-PAD IP underwent annual security audits by an independent security firm.

### Regulatory Oversight

Regulatory oversight of the APT Webstudy was provided by the University of Southern California Institutional Review Board (USC IRB). Regulatory oversight for the TRC was provided by Advarra, Inc., under a single IRB (sIRB) model. The sIRB model was established as a National Institute of Health (NIH) requirement for multi-site studies starting in 2018 to streamline the review of research that involves human subjects (18).

## Results

The APT Webstudy was launched on December 22, 2017, after a 6-month development period. The SRS and TRC EDC launched in May 2019. The TRC-PAD development team worked closely with the study team employing agile software development methods to design, build, test, and deploy these systems (19, 20).

**Figure 2.**
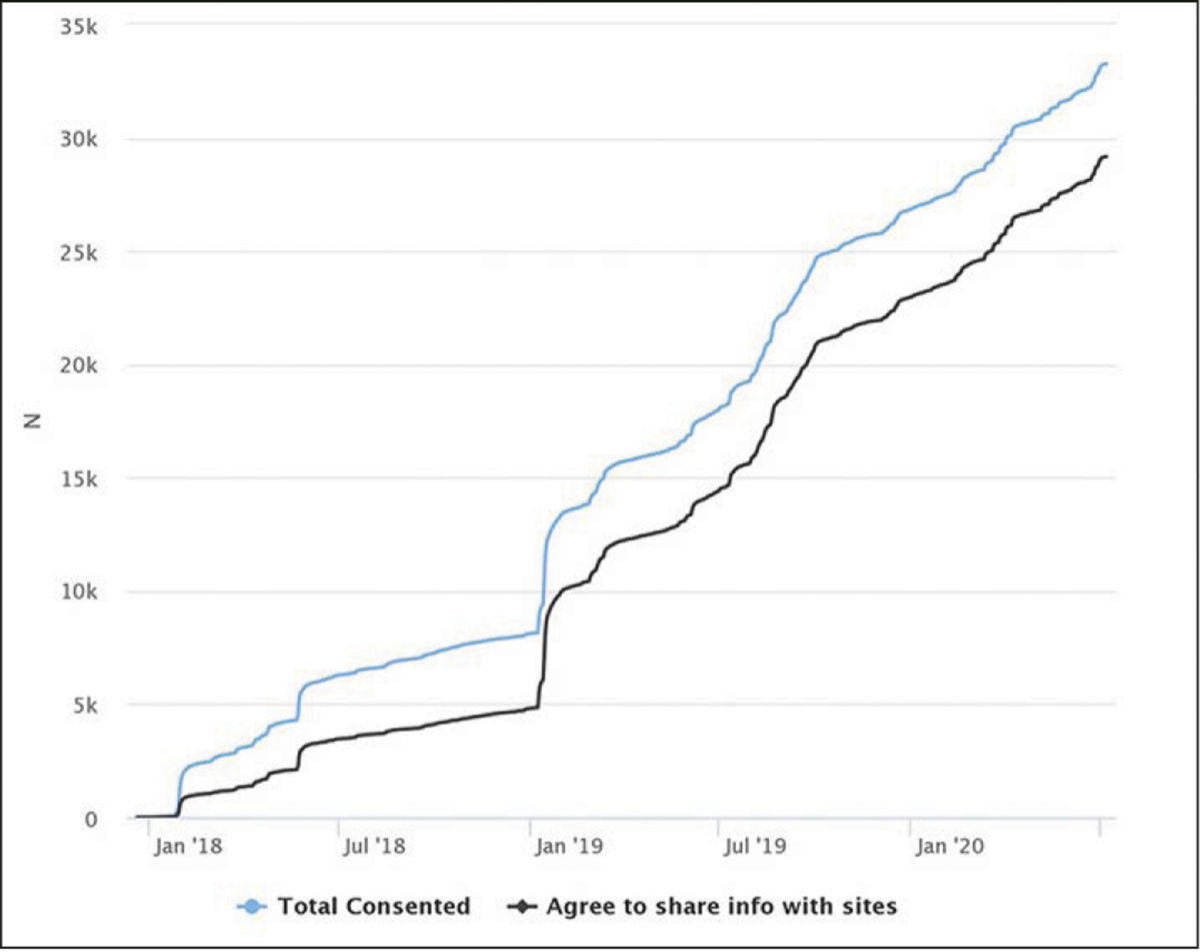
APT Webstudy Enrollment (December 22, 2017 to July 6, 2020)

As of July 6, 2020, 36,955 users had registered for an APT account. Of these registered users, 33,259 (90.0%) enrolled in the study via online consent and had completed more than 280,000 remote assessments (Figure 2). Recruitment into the APT Webstudy was driven by earned, owned, and shared media as well as feeder-based referrals. The APT cohort was geographically distributed across all 50 U.S. states, with participants concentrated in the coastal, midwestern, and southwestern states. Participant mobile device usage (43.0%) on the APT Webstudy was higher than initially expected. The demographic characteristics of the cohort are female (73.0%), non-Hispanic White (92.4%), with a mean age of 64.6 years (SD = 8.3). 87.7% agreed to have their contact information shared with the TRC sites. After one year of quarterly follow-up, 44.7% of participants were retained. The retention rate after two years of quarterly follow-up was 29.7%.

**Figure 3.**
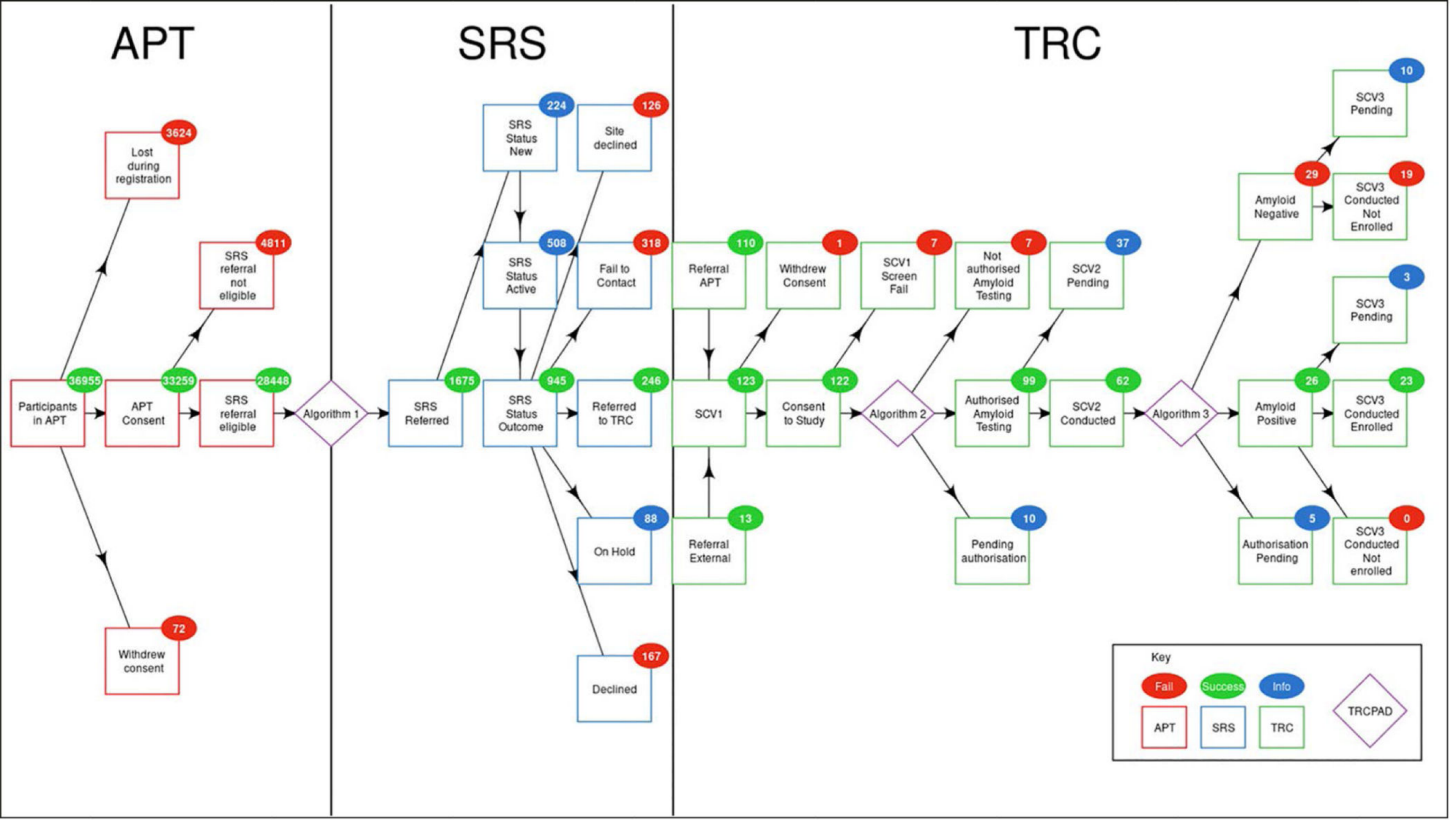
TRC-PAD Participant Flows by Stage (as of July 6, 2020)

TRC sites began in-person screening of participant referrals in August 2019. As of July 6, 2020, 27 of 35 TRC sites were activated and had received 1,675 risk-ranked participant referrals via the SRS. Of these, 246 (14.7%) participants were referred to the TRC for initial screening, 123 (50.0%) participants completed the initial screening visit, 99 (80.5%) participants were authorized for amyloid testing, 55 (55.6%) participants were biomarker-confirmed using amyloid PET or CSF assessment, 26 (47.3%) participants were found to be amyloid elevated, and 23 (88.5%) participants were enrolled into the TRC (Figure 3). The demographic characteristics of the standing TRC are female (51.2%), non-Hispanic White (94.2%), with a mean age of 72 years (SD = 7.8), and a mean SUVr of 1.14 (SD = 0.22). The median (interquartile range) cycle time from initial site referral via SRS to enrollment decision in the TRC was 28 (15 to 84) days. During this period, the TRC-PAD IP proved to be a scalable, cost-effective solution to support all stages of the TRC-PAD selection and recruitment.

## Discussion

Our early experiences with the TRC-PAD program and its underlying information management infrastructure, TRC-PAD IP, have demonstrated feasibility concerning the program aims. The TRC-PAD IP supported recruitment, screening, and enrollment of TRC-PAD participants by leveraging a secure, scalable, cost-effective cloud-based information technology (IT) architecture. The APT Webstudy has demonstrated effectiveness in terms of selecting and recruiting individuals from the general population who have an elevated risk of amyloid positivity. The APT Webstudy has proven effective in remotely assessing participants on a longitudinal basis. The SRS has demonstrated effectiveness in terms of allowing TRC sites to manage site referrals promptly. The TRC EDC has supported the coordination of the multi-stage risk-based screening and enrollment process into the standing TRC. Much work remains to demonstrate the effectiveness of the TRC-PAD program in accelerating recruitment into downstream AD clinical trials.

Several limitations should be considered. First, the TRC-PAD program has struggled to select and recruit a representative sample of participants across multiple sociodemographic dimensions (21). Efforts to address this challenge have included updating the APT Webstudy to support Spanish-speaking participants, however, more work is needed. Second, the architecture has proven to be resilient during a few web traffic spikes but has yet to be subjected to the type of surge (>10–100x daily web traffic) associated with an unexpected mention in a national media platform. Third, the integrations with third-party platforms, such as the Cogstate Brief Battery, have proven to be fragile, requiring frequent maintenance and technical support for participants. Addressing these technical issues may serve to improve both participant retention and risk algorithm predictive performance.

In May 2019, the TRC-PAD principal investigators selected the first set of downstream clinical trials slated to utilize the standing TRC to recruit participants. These clinical trials are scheduled to begin recruiting participants in North America (U.S. and Canada) in May 2020. The efficiencies that will accrue to these clinical trials are two-fold: 1) by drawing participants from the TRC, the expectation is that recruitment into these clinical trials will be accelerated by reducing the number of screen failures; and 2) the TRC clinical, neuropsychological, biofluid, and imaging data will be available to use as high-quality run-in data for downstream clinical trials (22). These combined efficiencies should yield significant savings both in terms of resources and time.

As the TRC-PAD program has been established over the past few years, promising new AD biomarkers have been developed. Plasma-based AD biomarkers, for example, have been shown to effectively predict amyloid positivity (23). When fully validated, the introduction of these diagnostic tools into the TRC screening process may be used to further increase effectiveness. The flexible and modular design of the TRC-PAD IP will accommodate the introduction of these new technologies.

Finally, as news of the TRC-PAD program’s progress has spread, a global network of programs modeled on its approach has begun to take shape. Investigators in several countries have been collaborating with the TRC-PAD program leadership to establish similar programs in their home regions. By using global cloud computing infrastructure and the TRC-PAD IP as a model architecture, these programs have been able to rapidly establish similar platforms in their local jurisdictions. Once fully operational, the global TRC-PAD network aims to provide AD clinical trials with a steady stream of well-characterized, biomarker-confirmed participants yielding savings in time, effort, and expense.

## Figures and Tables

**Table 1.** Technology Components of the TRC-PAD Informatics Platform

System	Applications/Tools	Cloud-based Infrastructure Services
APT Webstudy (APT)	Django	AWS
		- Elastic Beanstalk (EB)
		- RDS
		- S3
		Cogstate
		Google Analytics
		Mailchimp
Site Referral System (SRS)	Django	AWS
	Python	- Elastic Beanstalk (EB)
	ReactJS	- RDS
		- S3
		Google Analytics
		Mailchimp
TRC EDC	AngularJS	AWS
	Django	- Amazon Aurora
	Python	- Cognito
	ReactJS	- Elastic Beanstalk (EB)
		- S3
		Google Analytics
Analytics Platform (AP)	Python	AWS
	R	Lambda
		S3
		Shiny.io
		
IT Infrastructure	Docker	AWS
		- CloudFormation
		- CloudTrail
		- CloudWatch
		- Elastic Load Balancer (ELB)
		- Identity and Access Management (IAM)
		- Key Management Service (KMS)
		- Virtual Private Cloud (VPC)
Participant Technical Support		Google Voice
		Teamwork Desk
